# Higher efficiency of ISSR markers over plastid *psbA‐trnH* region in resolving taxonomical status of genus *Ocimum* L.

**DOI:** 10.1002/ece3.2483

**Published:** 2016-10-05

**Authors:** Amit Kumar, Priyanka Mishra, Kuppusamy Baskaran, Ashutosh K. Shukla, Ajit K. Shasany, Velusamy Sundaresan

**Affiliations:** ^1^ Department of Plant Biology and Systematics CSIR – Central Institute of Medicinal and Aromatic Plants, Research Centre Bengaluru India; ^2^ Biotechnology Division CSIR – Central Institute of Medicinal and Aromatic Plants Lucknow India

**Keywords:** ISSR, Lamiaceae, *Ocimum* spp., Phylogeny, *psbA‐trnH*

## Abstract

High level of morphological as well as chemical variability exists within the genus *Ocimum*, and its taxonomy and phylogenetic relationships are still doubtful. For evaluating interspecific genetic relationships among the *Ocimum* species, genotyping with intersimple sequence repeat (ISSR) markers and sequence analyses of noncoding *psbA‐trnH* intergenic region belonging to chloroplast DNA were carried out. Although ISSR markers are highly efficient and reproducible, they have not been used extensively in phylogenetic studies. The use of the plastidial barcode candidate was expected to provide more variable and informative insight into evolutionary rates, and was thus employed as a phylogenetic marker to assess interspecific relationships. This study revealed that the ISSR markers were more efficient than *psbA‐trnH* sequences in resolving the current status of *Ocimum* L. genus. Distance‐ and character‐based methodological approaches applied on the molecular data with biparental and maternal inheritance were used for deducing the phylogenetic relationships among *Ocimum* species. Average polymorphic information content (0.344) and resolving power (6.285) depicted through ISSR markers proved to be efficient in discriminating the studied species of *Ocimum*. The primers used in this study revealed 99.585% polymorphism across the species demonstrating the polymorphic nature of ISSR markers.

## 1 Introduction

The genus *Ocimum* L., belonging to family Lamiaceae (sixth largest family), collectively called as basil is an important economic and medicinal herb, widely distributed in tropical, subtropical, and warm temperate regions of the world (Paton, Harley, & Harley, 1999). Due to substantial taxonomical complexity caused by polyploidy and possibility of inter‐ and intraspecific hybridization within the genus *Ocimum*, estimates of the number of species vary from 30 (Paton, 1992) to 160 (Pushpangadan & Bradu, 1995). To add further to this complexity, there are varieties having similar chemical compositions within the species that do not differ significantly in morphology (Verma et al., 2016). Carvoic‐Stanko et al. (2010) reported that basic chromosome number in *Ocimum* spp. is *x* = 12. *O. basilicum* and *O. americanum* are reported to be tetraploid (2*n* = 4*x* = 48) and hexaploid (2*n* = 6*x* = 72), respectively. *O. tenuiflorum* is a perennial shrub with a basic chromosome number of *x* = 9, while *O. gratissimum* has a basic chromosome number *x* = 10. *O. tenuiflorum* L., *O. basilicum* L., *O. gratissimum* L., *O. kilimandscharicum* Gurke, *O. americanum* L., and *O. africanum* Lour. are important species belonging to the genus *Ocimum* that grow in different parts of the world and are known for their diverse medicinal properties and as sources of important essential oils (Carvoic‐Stanko et al., 2010; Prakash & Gupta, 2005). In India, nine species are found, of which three are exotic (Balyan & Pushpangadan, 1988). *O. adscendens* is a lesser known species with few studies carried out on it (Asthana, Tripathi, & Dixit, 1986; Verma et al., 2016).

*Ocimum* spp. have been used in folk medicine due to their diverse biological activities, such as mosquito repellency, antimicrobial, insecticidal, antiseptic, carminative, stimulant, antispasmodic, antioxidant, and antipyretic properties (Padalia, Verma, Chauhan, & Chanotiya, 2013). *O. tenuiflorum* has been well documented in Ayurveda for its therapeutic potential as a *Kapha*‐balancing plant (Shukla et al., 2013) and possesses antioxidant and antimicrobial activities due to its phenolic and aromatic compounds (Verma, Padalia, Chauhan, & Thul, 2013). The oil components of *Ocimum* have been found to be produced by two different biochemical pathways, viz. shikimic acid pathway (phenylpropanoids) and mevalonic acid pathway (terpenes). Essential oil consisting of monoterpenes, sesquiterpenes, and phenylpropanoids reported from *Ocimum* spp. includes linalool, linalyl acetate, geraniol, citral, camphor, eugenol, methyl eugenol, methyl chavicol, methyl cinnamate, thymol, safrole, which are of immense value and have been used principally in the food and cosmetic industries (Balyan & Pushpangadan, 1988; Vina & Murillo, 2003).

The genus *Ocimum* is very variable and possesses a wide range of intra‐ and interspecific genetic diversity. According to previous reports on *Ocimum*, the genus is grouped into three sections: *Ocimum* (*Ocymodon* Benth.), *Hierocymum* Benth., and *Gymnocymum* Benth. Section *Ocimum* was again divided into subsections: *Ocimum*,* Gratissima*, and *Hiantia* Benth (Paton et al., 1999). Further, Pushpangadan divided the whole taxa into two groups: the Basilicum group, containing herbaceous annuals/perennials with black, ellipsoid, strongly mucilaginous seeds, and the Sanctum group, consisting of perennial shrubs with brown, globose, nonmucilaginous, or weakly mucilaginous seeds. According to Pushpangadan's classification, the Basilicum group contains only section *Ocimum* subsection *Ocimum* (Carvoic‐Stanko et al., 2010). However, Pushpangadan's infrageneric classification does not act in accordance with the *International Code of Botanical Nomenclature*. Several studies throughout worldwide related to cytotaxonomical studies, taxonomical status and geographic distribution, classification and relationships, genetic diversity and phylogenetic study, and chemical characterization (Balyan & Pushpangadan, 1988; Carvoic‐Stanko et al., 2011; Paton et al., 1999; Sobti & Pushpangadan, 1979; Verma et al., 2016; Vieira & Simon, 2000) reported ample extent of genetic variability within the genus *Ocimum,* making it taxonomically and phylogenetically convoluted.

Inter simple sequence repeat (ISSR) markers (Zietkiewicz, Rafalski, & Labuda, 1994) are dominant genetic markers generated through polymerase chain reaction (PCR) amplification of genomic DNA using a single primer that amplifies the regions between adjacent, inversely oriented simple sequence repeats, provided the primer binding sites are located within an amplifiable range. The evolutionary rate of change within microsatellites is considerably higher as compared to most other types of DNA, which increases the likelihood of polymorphism in these sequences. However, ISSR markers are present in both nuclear and organellar genomes and are responsible for the generation of highly polymorphic loci. ISSR markers have frequently been used in the characterization of germplasm collections and in the screening of genetic diversity within species (Kumar, Mishra, Singh, & Sundaresan, 2014; Mishra et al., 2015), cultivar identification (Chowdhury, Vandenberg, & Warkentin, 2002), molecular mapping, genomic instability assessment, but they are rarely used for phylogeny reconstruction at the species level. The ISSR technique is less expensive comparatively to RFLPs and AFLPs, giving higher reproducibility than RAPDs. Individuals of the same species usually show a few to no difference between their ISSR patterns, whereas closely related taxa, that is, subspecies and species, give a specific banding profile that can be used for deducing phylogenetic relationship.

Chloroplast DNA (cpDNA) is the smallest as compared to mitochondria and nuclear genome, and during the course of evolution, it is assumed to be conserved in terms of nucleotide substitution with very little rearrangements, which makes the molecule best suited to be used in resolving phylogenetic relationships especially at higher levels of evolution (Zurawski & Clegg, 1987). cpDNA sequences, particularly the noncoding regions, were in demand as source of markers because they are evolving faster as they have fewer functional constraints than gene‐coding regions in terms of nucleotide substitution rates (Kimura, 1983). Also, this allows comparisons between seed gene flow and pollen gene flow as well as the identification of hybridization events when compared to nuclear, biparentally inherited DNA, demonstrating genealogical structure (Dong et al., 2013). Thus, noncoding cpDNA sequences are expected to provide more variable and informative characters for phylogenetic studies of evolutionary rates. The *psbA–trnH* intergenic spacer, an evolutionarily plastid region, is employed as a phylogenetic marker to assess interspecific relationships.

*Ocimum* species are similar in apparent vegetative morphology and often debated upon with regard to their nomenclature and taxonomical positioning. The nomenclature of *Ocimum* species and varieties is complicated and often confusing. Besides, different physicochemical properties have been observed in the oils extracted from morphologically identical plants (single phenotype) (Singh et al., 2004). This study on *Ocimum* species from India was carried out with the following objectives: (1) comparative phylogenetic analysis using ISSR markers and plastid DNA (*psbA‐trnH*) sequences and (2) evaluation of the efficiency of ISSR markers vis‐à‐vis chloroplast noncoding *psbA‐trnH* region in elucidating the phylogenetic relationship among medicinally/agro‐economically important species of *Ocimum*.

## 2 Materials and Methods

### 2.1 Plant material

**Figure 1:**
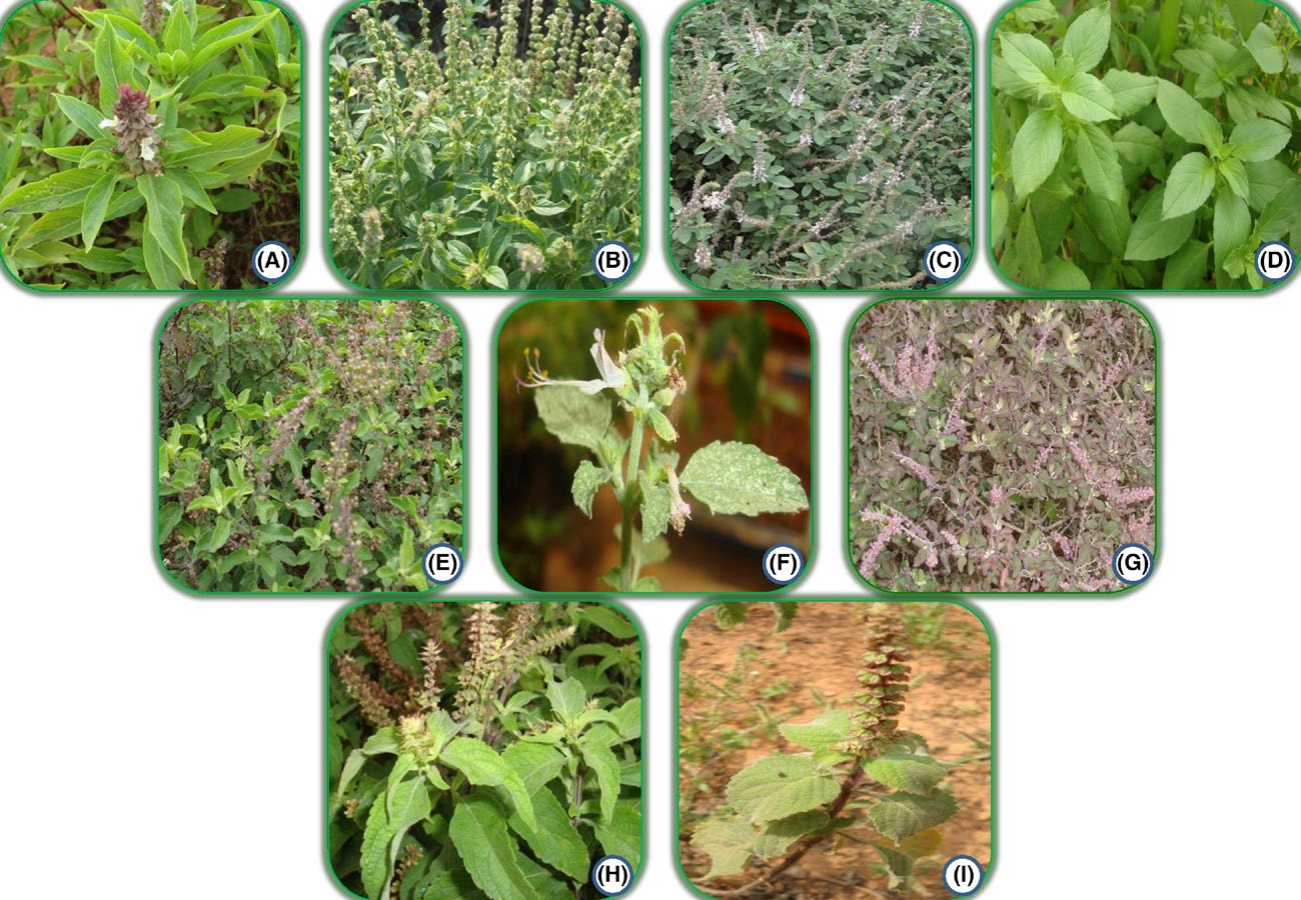
Different *Ocimum* species from India. (A) *Ocimum basilicum*, (B) *Ocimum americanum*, (C) *Ocimum kilimandscharicum*, (D) *Ocimum africanum*, (E) *Ocimum tenuiflorum* var. Ram tulsi, (F) *Ocimum adscendens*, (G) *Ocimum tenuiflorum* var. Krishna tulsi, (H) *Ocimum gratissimum*, (I) *Ocimum viride*

Seeds from seven different species of *Ocimum* (few of them represented by different varieties) were collected from different parts of India (Figure 1; Table 1). Seeds were grown in the ChemBio genebank at CSIR‐CIMAP Research Centre, Bengaluru, India. The different *Ocimum* species included were *O. tenuiflorum* L.*, O. basilicum* L.*, O. africanum* Lour, *O. americanum* L.*, O. kilimandscharicum* Gurke*, O. gratissimum* L.*, O. viride* Willd., and *O. adscendens* Willd. Along with the *Ocimum* species, an outgroup taxon *Pogostemon cablin* L. was chosen because of its known distant relationship. According to the plant list, *O. viride* is synonymous to *O. gratissimum* (http://www.theplantlist.org/), but looks morphologically different. All the voucher specimens of the plant accessions used were deposited in the herbarium at CSIR‐Central Institute of Medicinal and Aromatic Plants, Research Centre, Bengaluru, India, located at 12°58′N latitude and 77°35′E longitude for future reference.

**Table 1:** Geographic location of *Ocimum* seed collections

S. no.	Code	Species	Place of collection	State	Latitude	Longitude
1	V1	*Ocimum tenuiflorum* var. Ram tulsi	Venkateswarapuram	Tamil Nadu	N08°55′	E77°34′
2	V2	*Ocimum tenuiflorum* var. Krishna tulsi	Rasipuram	Tamil Nadu	N11°28′	E78°09′
3	V3	*Ocimum basilicum*	Hilsa	Bihar	N25°20′	E85°17′
4	V4	*Ocimum africanum*	Kanyakumari	Tamil Nadu	N08°04′	E77°32′
5	V5	*Ocimum basilicum* var. purpurascens	Vasudevanallur	Tamil Nadu	N09°14′	E77°24′
6	V6	*Ocimum americanum*	Vellur	Tamil Nadu	N11°06′	E78°00′
7	V7	*Ocimum kilimandscharicum*	Yercaud	Tamil Nadu	N11°46′	E78°12′
8	V8	*Ocimum gratissimum*	Yercaud	Tamil Nadu	N11°46′	E78°12′
9	V9	*Ocimum adscendens*	Rajapalayam	Tamil Nadu	N09°28′	E77°33′
10	V10	*Ocimum viride*	Jammu Tawi	Jammu & Kashmir	N32°43′	E74°50′
11	V11	*Pogostemon cablin*	Bangalore	Karnataka	N12°58′	E77°35′
12	V12	*Ocimum tenuiflorum* var. CIM‐Ayu	Lucknow	Uttar Pradesh	N26°53′	E80°58′
13	V13	*Ocimum tenuiflorum* var. CIM‐Angana	Lucknow	Uttar Pradesh	N26°53′	E80°58′
14	V14	*Ocimum basilicum* var. CIM‐Saumya	Lucknow	Uttar Pradesh	N26°53′	E80°58′

### 2.2 DNA isolation

Fresh plant leaves were collected, and genomic DNA was isolated using the protocol described by Khanuja, Shasany, Darokar, and Kumar (1999). The purity and concentration of isolated genomic DNA were quantified spectrophotometrically (NanoDrop ND‐1000, Nanodrop Technologies, Wilmington, Delaware, USA) by measuring absorbance at 260 nm and 280 nm for the OD_260_/OD_280_ ratio. Its integrity was checked by visualizing it under UV light after electrophoresis on 0.8% agarose gel. Stock DNA was diluted to make a required working solution of 25 ng/μl.

### 2.3 ISSR‐PCR amplification

Twenty ISSR primers were procured from the University of British Columbia (UBC set No. 9). PCR reaction was prepared in a total volume of 25 μl and contained 1 ×  Taq DNA polymerase buffer (with MgCl_2_) (GeNei™), 50 ng genomic DNA, 1 unit of Taq DNA polymerase (GeNei™), 200 μmol/l of each dNTP (dATP: dTTP: dCTP: dGTP in 1:1:1:1 parts) (GeNei™), and 0.5 μmol/l primer. Amplifications were carried out using a thermal cycler (iCycler™ Thermal Cycler, Bio‐Rad, USA) using the following parameters: initial denaturation at 94°C for 5 min; 45 cycles of denaturation at 94°C for 1 min, annealing at 48–60°C (Table 2) for 1 min, extension at 72°C for 2 min; final extension at 72°C for 7 min. Amplicons were electrophoresed on 1.2% agarose gel alongside a 2‐Log DNA ladder (New England Biolabs Inc.). Gel photographs were taken by means of Bio‐Rad Universal Hood II gel documentation system (Bio‐Rad laboratories Inc.).

**Table 2:** Marker parameters calculated for each ISSR primer used with *Ocimum* spp

S. No.	Primer	Sequence (5′–3′)	GC%	*T* [°C]	TB	PB	PPB (%)	PIC	RP
1	UBC 807	(AG)_8_T	47	52	12	12	100	0.374	6.571
2	UBC 809	(AG)_8_G	53	60	14	14	100	0.333	6.571
3	UBC 810	(GA)_8_T	47	52	16	16	100	0.325	7.857
4	UBC 823	(TC)_8_C	53	54	14	14	100	0.341	7.142
5	UBC 825	(AC)_8_T	47	60	10	10	100	0.307	4.428
6	UBC 826	(AC)_8_C	53	52.5	6	6	100	0.229	1.857
7	UBC 828	(TG)_8_A	47	52	12	12	100	0.363	6.285
8	UBC 834	(AG)_8_YT	47	52.5	14	14	100	0.382	8.714
9	UBC 835	(AG)_8_YC	53	52	14	14	100	0.365	8.142
10	UBC 841	(GA)_8_YC	53	52	8	8	100	0.395	4.857
11	UBC 843	(CT)_8_RA	47	53	6	6	100	0.299	2.571
12	UBC 844	(CT)_8_RC	53	54	10	10	100	0.357	5.999
13	UBC 845	(CT)_8_RG	53	53	13	13	100	0.342	6.571
14	UBC 848	(CA)_8_RG	53	52.5	13	13	100	0.368	7.142
15	UBC 855	(AC)_8_YT	47	53	8	8	100	0.376	4.428
16	UBC 856	(AC)_8_YA	47	53	16	16	100	0.343	7.999
17	UBC 862	(AGC)_6_	67	57.2	13	12	92.30	0.328	6.285
18	UBC 866	(CTC)_6_	67	57.2	14	14	100	0.338	7.142
19	UBC 876	(GATA)_2_(GACA)_2_	38	48	13	13	100	0.397	8.285
20	UBC 881	(GGGTG)_3_	80	58.7	15	15	100	0.327	6.857
**Total**					**241**	**240**			
	Avg./Primer				**12.05**	**12.00**	**99.585**	**0.344**	**6.285**

*T* [°C], annealing temperature; R, (A, G); Y, (C, T); TB, total band; PB, polymorphic band; PPB (%), percentage polymorphic band (%); PIC, polymorphic information content; RP, resolving power of primer.

### 2.4 PCR amplification and sequencing of *psbA‐trnH* (cpDNA)

Noncoding *psbA‐trnH* region of cpDNA was amplified using universal primers fwd‐PA: 5′‐GTT ATG CAT GAA CGT AAT GCT C‐3′ and rev‐TH: 5′‐CGC GCA TGG TGG ATT CAC AAT CC‐3′ (Newmaster & Ragupathy, 2009; Zheng, Jiang, Wu, Wang, & Huang, 2014). PCR was run in a manner similar to that of ISSR‐PCR with the following cycling parameters: an initial denaturation at 94°C for 5 min; 30 cycles of denaturation at 94°C for 1 min, annealing at 55°C for 1 min, extension at 72°C for 90 s; final extension at 72°C for 7 min. PCR products were visualized on a 1.2% agarose gel and purified using QIAquick PCR purification kit (QIAGEN) prior to sequencing in both directions on an ABI 3130XL automated sequencer (Applied Biosystems) using both the primers. Sequences generated were deposited at the GenBank (NCBI) (Table 3).

**Table 3:** Characteristics of *Ocimum* spp. sequences (of noncoding *psbA‐trnH* region) deposited in GenBank (NCBI)

S. no.	Species		GenBank accession no.	Length	GC (%)
1	*Ocimum tenuiflorum* var. Ram tulsi	Forward	KT338799.1	328	28.04
		Reverse	KU666421.1	372	29.30
2	*Ocimum tenuiflorum* var. Krishna tulsi	Forward	KT338800.1	328	28.04
		Reverse	KU666422.1	321	28.97
3	*Ocimum basilicum*	Forward	KT338801.1	323	27.55
		Reverse	KU666423.1	368	29.61
4	*Ocimum africanum*	Forward	KT338802.1	338	28.69
		Reverse	KU707910.1	321	26.79
5	*Ocimum basilicum* var. purpurascens	Forward	KT338803.1	323	27.24
		Reverse	KX096039.1	310	30.32
6	*Ocimum americanum*	Forward	KT338804.1	343	28.86
		Reverse	KU707911.1	284	26.40
7	*Ocimum kilimandscharicum*	Forward	KT338805.1	324	28.39
		Reverse	KU707912.1	292	26.02
8	*Ocimum gratissimum*	Forward	KT338806.1	320	29.68
		Reverse	KX013103.1	368	29.61
9	*Ocimum adscendens*	Forward	KT338807.1	328	28.65
		Reverse	KX013104.1	373	29.22
10	*Ocimum viride*	Forward	KT338808.1	320	28.75
		Reverse	KX013105.1	358	30.72
11	*Pogostemon cablin*	Forward	KT338809.1	187	29.41
		Reverse	KX013106.1	271	30.62
12	*Ocimum tenuiflorum* var. CIM‐Ayu	Forward	KT338810.1	328	28.04
		Reverse	KX096040.1	373	29.49
13	*Ocimum tenuiflorum* var. CIM‐Angana	Forward	KT338811.1	328	28.65
		Reverse	KX096041.1	372	30.37
14	*Ocimum basilicum* var. CIM‐Saumya	Forward	KT338812.1	343	28.86
		Reverse	KX096042.1	344	28.19

### 2.5 Data analysis

Amplified ISSR products were manually scored for band presence (1) or absence (0) for each accession, and a binary data matrix was constructed. Primer banding characteristics such as the number of total bands (TB), the number of polymorphic bands (PB), and the percentage of polymorphic bands (PPB) were obtained. Polymorphic information content (PIC) analysis is useful for selection of the most appropriate marker for genetic mapping and phylogenetic analysis. It provides an estimate of the discriminating power of the marker based on the number of noticeable alleles and their distribution (Mishra et al., 2015; Powell et al., 1996). Resolving power (RP) of a marker is yet another parameter for checking the efficiency of discrimination potential of any marker system (Kumar et al., 2014; Prevost & Wilkinson, 1999).

To check the genetic relatedness among the *Ocimum* spp., data matrix of ISSR markers was converted into genetic similarity matrix using Jaccard coefficient (Jaccard, 1908) in SPSS 17.0 (SPSS Inc.) and NTSYS‐PC 2.02j (Rohlf, 1998). Bootstrap analysis (Felsenstein, 1985) using Nei's (1973) genetic distance search with 1,000 replicates was performed to obtain the confidence of the neighbor‐joining tree using the software TREECON (Van De Peer & Wachter, 1994). Further, to highlight the resolving power of the ordination, principal component analysis (PCA) was performed based on similarity coefficient of data realized from ISSR average similarity indices using SPSS statistics 17.0 software (SPSS Inc.).

### 2.6 Sequence alignment and phylogenetic analysis

Forward and reverse electropherograms were superimposed, visually checked, and edited using the BioEdit software (http://www.mbio.ncsu.edu/BioEdit/bioedit.html). Sequence identity was confirmed by comparison with sequences deposited in public databases, using the BlastN search algorithm (http://www.ncbi.nlm.nih.gov/BlastN). Multiple sequence alignment of *psbA‐trnH* region sequences was performed using clustalW.

Neighbor‐joining (NJ) phylogenetic trees were constructed using Kimura 2‐parameter (K2P) model, and reliability of the trees was tested with the 1000 replicates of nonparametric bootstrap (Felsenstein, 1985) for estimating evolutionary distances using software TREECON (Van De Peer & Wachter, 1994). Molecular phylogeny trees were reconstructed for character support using maximum parsimony (MP) and maximum likelihood (ML) through phylogeny program PAUP*4.0b10 (Swofford, 2002). For MP, all character transformations were weighted equally, and gaps were treated as missing data. Tree Bisection and Reconnection (TBR) branch swapping option, addition sequence option “random,” and MAXTREES set to auto increase, and 100 heuristic searches per replicate were applied. For ML, best‐fit nucleotide substitution model was tested to facilitate comparisons between 56 alternative models in PAUP*4.0b10. Heuristic search was conducted under the general time‐reversible (GTR) and gamma distributed with invariant sites (G + I) model with TBR branch swapping algorithm on a starting tree built under the parsimony criterion and bootstrapped with 1,000 replicates. Estimated substitution rate matrix: AC = 1.4792878; AG = 2.155157; AT = 0.54992304; CG = 1.2440845; CT = 1.0431192; GT = 1.000000. Gamma shape parameter = 0.678. Assumed proportion of invariable sites = 0.2897. MP and ML trees were visualized and saved using FigTree 1.4.2 (Rambaut, 2014).

## 3 Results

### 3.1 Performance of ISSR markers

The GC percentages of the primers used in this study were within the range 38%–80% (with eight primers having 47% GC content and another eight having 53% GC content). Eleven of the primers were 18 bp long, seven were 17 bp long, and one each was 16 and 15 bp long. All the used dinucleotide primers were anchored at their 3′‐end with one or two degenerate nucleotide(s), to increase the specificity and polymorphism. Twenty ISSR primers used in this study yielded 241 loci ranging from 0.2 to 1.5 kb, out of which 240 loci were polymorphic in nature representing 99.585% polymorphism. The number of loci reproduced by these arbitrary primers was found to range from 6 to 16 of different sizes with an average frequency of 12.05 loci per ISSR primer. The primers UBC 810 and UBC 856 that gave the maximum number of amplicons (16) were the most informative, while the primers UBC 826 and UBC 843 that gave the minimum number of products (6) were least informative. A total of 1,040 clear DNA fragments were scored from all *Ocimum* species and its varieties including outgroup taxa *P. cablin*. Two hundred and forty variable loci showed fragment frequency range of 0.071–0.928. Of these, 113 loci (47.08%) showed a frequency __0.25, whereas 99 (41.25%) of the loci showed a frequency range of ≥0.25 to ≤0.5, and 28 (11.67%) showed a frequency >0.5. The ISSR loci were 100% polymorphic at species level for each primer except UBC 862 (Table 2). In this study, the highest PIC value of 0.397 was observed for primer UBC 876, and the lowest PIC value of 0.229 was observed for primer UBC 826, with an overall average PIC value of 0.344 per primer. The highest RP value was observed with the primer UBC 834 (8.714) and the lowest with the primer UBC 826 (1.857) with an average RP of 6.285 per primer.

### 3.2 *Cluster analysis based on the ISSR*‐*derived genotyping*

**Figure 2:**
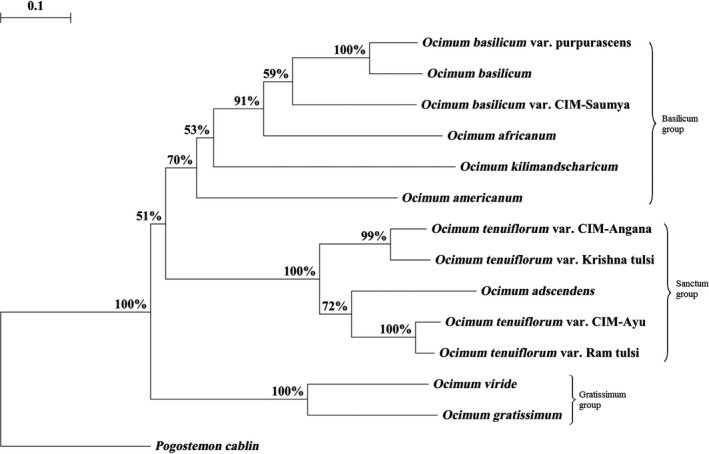
Neighbor‐joining bootstrap tree resulting from ISSR dataset. Bootstrap value less than 50% is omitted

**Figure 3:**
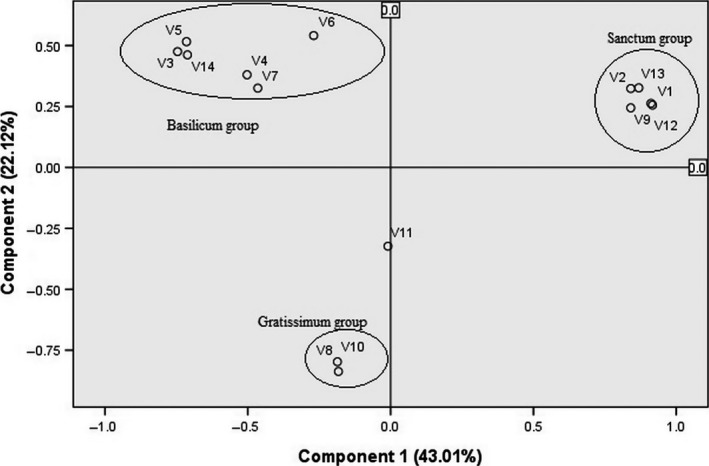
Principal component analysis (PCA) of *Ocimum* ISSR data. These cumulatively account for 65.17% (43.05% and 22.12%, respectively) of the data variance, the third axis (not shown) representing 9.13%; V1–V14 represent different plant genotypes as listed in Table 1

Fingerprinting dataset composed of 241 ISSR loci with 1040 DNA fragments was selected for banding pattern similarity analysis based on Jaccard coefficient. The pairwise genetic distance based on the similarity matrix ranged from 0.098 (*O. tenuiflorum* var. Ram tulsi and *O. tenuiflorum* var. CIM‐Ayu) to 0.888 (*O. kilimandscharicum* and *O. gratissimum*) with an average pairwise genetic distance of 0.493. Genetic relationships were determined by neighbor‐joining tree based on the Nei's genetic distance obtained from the ISSR profiles and showed two major clusters with 51% bootstrap value, one of “Basilicum” group and other of “Sanctum” group, while *P. cablin* was placed as an outgroup as expected. “Gratissimum” group was bifurcated from “Basilicum” and “Sanctum” groups with 100% bootstrap value. “Basilicum” group consisted of *O. americanum*,* O. kilimandscharicum*,* O. africanum*, and *O. basilicum* with its varieties. “Sanctum” group consisted of *O. adscendens* and *O. tenuiflorum* with its varieties. The phylogram depicted in Figure 2 clearly shows that *O. gratissimum* and *O. viride* diverge earlier than the “Basilicum” and “Sanctum” groups. Principal component analysis (PCA) derived on the basis of ISSR data is illustrated in Figure 3. The first two principal component axes accounted for 43.01% and 22.12% data variance, respectively, representing 65.17% of data variance cumulatively, which is sufficient to resolve the taxa into distinct groups. The third (9.13%) and fourth (6.72%) axes of the data variance did not significantly improve the resolution of the taxa. Groupings with the PCA analysis are in agreement with the NJ tree as revealed by the TREECON software.

### 3.3 *psbA‐trnH* sequence data analysis

Chloroplast *psbA‐trnH* intergenic spacers were successfully amplified in all *Ocimum* species and *P. cablin*. Forward sequences of *psbA‐trnH* region were observed in the range of 320 to 343 bp for *Ocimum* species and 187 bp for *P. cablin*. The average GC content in the sequences was low (28.49%). The aligned sequence length of *psbA‐trnH* was 357 bp, harbored 310 (86.83%) characters constant, 29 (8.12%) variable sites but parsimony‐uninformative and 18 (5.04%) parsimony‐informative sites. Nucleotidic divergence (p‐distance), including the outgroup, ranged between 0.00 and 0.155, with an average of 0.025. For *Ocimum* species, the maximum pairwise p‐distance of 0.039 was observed with *O. adscendens* and *O. basilicum* and with *O. adscendens* and *O. basilicum* var. purpurascens.

**Figure 4:**
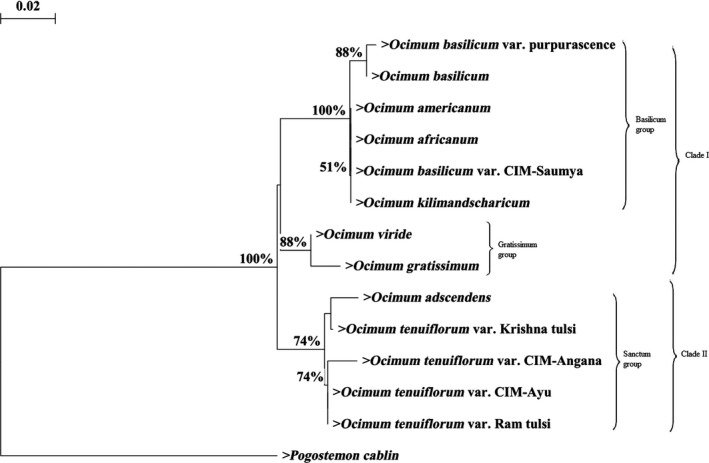
Neighbor‐joining bootstrap tree using Kimura 2‐parameter algorithm. Phylogram resulting from *psbA‐trnH* nucleotide sequence data

**Figure 5:**
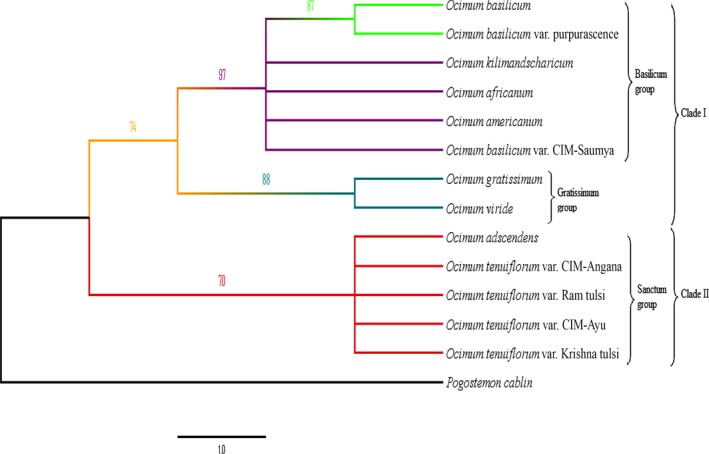
Equally weighted parsimony tree of *psbA‐trnH* sequence data for *Ocimum* species. Single consensus parsimonious trees (tree length = 54, CI = 0.926, RI = 0.934); values above branches are bootstrap value of 1,000 replicates; branch colors represent the support (violet = maximum; red = minimum)

**Figure 6:**
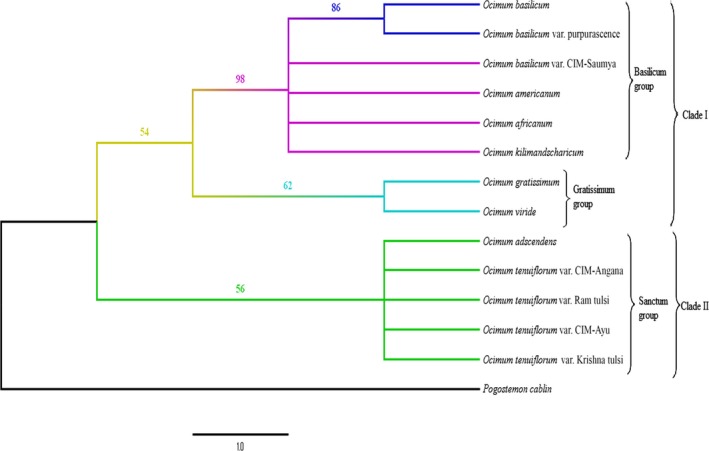
Reconstruction of a phylogenetic tree from *psbA‐trnH* sequence dataset by maximum likelihood using GTR + G + I model of substitution. Bootstrap support values of 1,000 replicates are given above the branches. Branch colors represent the support (violet = maximum; red = minimum)

Dataset of *psbA‐trnH* sequences was used to reconstruct NJ, MP, and ML trees for 14 genotypes comprising of eight *Ocimum* species (few of them represented by different varieties) and also including an outgroup *P. cablin*. The topology of all the NJ (Figure 4), MP (Figure 5), and ML (Figure 6) trees is almost congruent and is supported by bootstrap value of more than 50%. Topology of MP and ML tree constructed through the program PAUP*4.0b10 is similar with different bootstrap value. In the MP analysis, after bootstrapping the single most parsimonious tree was retained with a tree length of 54, a consistency index (CI) of 0.926, a retention index (RI) of 0.934, and a rescaled consistency index (RC) of 0.865. A single best tree with likelihood –ln L = 736.44021 was found in ML analyses. The trees show that “Gratissimum” group is sister to “Basilicum” group with support value of NJ <50%, MP 74%, and ML 54%.

## 4 Discussion

Several groups have been working worldwide on *Ocimum* species targeting different aspects and using distinct tools. Some of these are as follows: taxonomical status and geographic distribution (Balyan & Pushpangadan, 1988); cytotaxonomical studies (Sobti & Pushpangadan, 1979); classification and relationships (Paton et al., 1999); genetic diversity and phylogenetic study using different molecular markers (Lal, Mistry, Thaker, Shah, & Vaidya, 2012; Patel, Fougat, Kumar, Mistry, & Kumar, 2014; Singh et al., 2004); chemical characterization (Carvoic‐Stanko et al., 2011; Verma et al., 2016; Vieira & Simon, 2000); compositional diversity in essential oils (Verma et al., 2013); genetic relations based on molecular markers, nuclear DNA content, and chromosome number (Carvoic‐Stanko et al., 2010); taxonomical distribution, medicinal properties, and drug development potentiality (Nahak, Mishra, & Sahu, 2011); nucleotide‐based validation by evaluating three candidate barcodes of the chloroplast region (Christina & Annamalai, 2014). However, no report is found on comparative phylogenetic study in *Ocimum* using PCR (ISSR)‐based and sequence (cpDNA)‐based analyses. To the best of our knowledge, this is the first report on the comparative phylogenetic study of *Ocimum* species through ISSR markers and plastid DNA region. The phylogenetic relationships of *Ocimum* species were inferred using distance (NJ)‐based approach applied on ISSR genomic data as well as both distance‐ and character‐based (MP and ML) methodological approaches applied on *psbA‐trnH* region sequence data.

In the present study, 14 genotypes belonging to seven species of *Ocimum* (few of them represented by different varieties) and also including one outgroup *P. cablin* were studied using ISSR markers. The selection of ISSR markers was based on their relative technical simplicity, higher level of polymorphism detected, robustness, cost effectiveness (less time consuming and less expensive), easily adaptability to any plant species, and capability to target sequences that are abundant throughout the eukaryotic genome. The polymorphism level of 99.585% observed through ISSR analysis in the present study was in line with the earlier studies carried out by Patel et al. (2014), whereby ISSR analysis of five *Ocimum* species showed 98.17% polymorphism. Chen et al. (2013) found 97% polymorphism in four *Ocimum* species, and Lal et al. (2012) found 100% polymorphism in six species of *Ocimum*. High polymorphism of ISSR over other marker systems was also reported in many previous studies, such as in *Ocimum* (Chen et al., 2013; Lal et al., 2012), *Allium* (Mukherjee et al., 2013), and *Arachis* (Raina et al., 2001). The average PIC value of 0.344 was observed in this study, which was closer to 0.5, the maximum value of PIC for any dominant marker (Nagy et al., 2012). Although ISSR amplifies the regions between two microsatellites, the average PIC of ISSR marker systems was reasonably higher. The high PIC value obtained for the ISSRs reflects the efficiency of the marker to simultaneously analyze a large number of bands, rather than the level of polymorphism detected (Powell et al., 1996). High levels of polymorphism and PIC found in the present work showed that ISSR markers are suitable for differentiating the genus *Ocimum* and its closely related varieties. Prevost and Wilkinson (1999) reported that resolving power (RP) of a primer correlates strongly with its ability to distinguish between genotypes. In other words, RP provides quantitative data allowing comparisons between primers, including those that are able to distinguish all genotypes under examination. It can also be used to predict the performance of groups of primers. In the present study, average RP value of 6.285 proved the efficiency of ISSR markers to discriminate among the *Ocimum* species.

ISSR analyses based on the distance‐based (NJ) method grouped the *Ocimum* spp. in three groups: “Basilicum,” “Sanctum,” and “Gratissimum” groups. This grouping is explained by the earlier studies carried out by Paton et al. (1999) based on morphological characters, which grouped *Ocimum* into sections and subsections. Sect. “*Ocimum*” subsect. “*Ocimum*” comprised of “Basilicum” group, and sect. “*Ocimum*” subsect. “*Gratissima*” comprised of “Gratissimum” group, while *O. tenuiflorum* is placed in section “*Hierocymum*” subsect. “*Foliosa*.” Moreover, *O. gratissimum* (2*n* = 40) and *O. tenuiflorum* (2*n* = 36) have a smaller number of chromosomes compared to *O. basilicum* (2*n* = 48) and *O. americanum* (2*n* = 72) (Carvoic‐Stanko et al., 2011). Carvoic‐Stanko et al. (2010) also reported the same type of grouping based on molecular markers, nuclear DNA content, and chromosome number. Our studies clearly demonstrated that morphologically dissimilar species *O. gratissimum* and *O. viride* form a single group. “Gratissimum” group is bifurcated earlier with 100% support value from “Basilicum” and “Sanctum” group in ISSR–NJ tree, which is supported by the study carried out by Carvoic‐Stanko et al. (2011). According to the results presented, *O. kilimandscharicum* grouped together with *O. basilicum*,* O. africanum*, and *O. americanum*, which is again in agreement with the study done by Khosla (1995). Paton (1992) placed *O. kilimandscharicum, O. basilicum*, and *O. americanum* in section “*Ocimum*” subsection “*Ocimum*,” which also backed the same grouping pattern. *O. kilimandscharicum* was found to group with *O. basilicum* in an earlier rapid amplification of polymorphic DNA (RAPD)‐based study also (Shasany, Shukla, & Khanuja, 2007).

Multivariate approach was used to complement the information retrieved from the cluster analysis methods because it is more informative regarding distances among major groups (Taran, Zhang, Warkentin, Tullu, & Vanderberg, 2005). PCA analysis used in the present study provides complementary information to the cluster analysis, as it allows a graphical presentation of *Ocimum* species in a scatter plot. The PCA of *Ocimum* species and an outgroup *P. cablin* explained 74.3% of total variation by the first three principal components. Grouping in PCA based on ISSR marker dataset corresponded well to the ISSR–NJ phylogenetic tree.

On the other hand, distance‐based (NJ) and character‐based (MP and ML) trees depicted through *psbA‐trnH* sequence dataset grouped the *Ocimum* species into two major clades. Clade I indicated that the “Gratissimum” group was sister to “Basilicum” group with reliable bootstrap values (NJ <50%, MP 74%, ML 54%). Clade II was comprised of *O. tenuiflorum* with its varieties and *O. adscendens*. The CI (0.926) and RI (0.934) of the *psbA‐trnH* consensus maximum‐parsimony tree are high, suggesting that the phylogenetic reconstruction is fairly reliable. Unlike the present grouping, many workers reported grouping of “Gratissimum” group with “Sanctum” group on the basis of morphological, cytological, and oil characters (Khosla, 1995; Sobti & Pushpangadan, 1979) or genetic similarity defined through RAPD (Singh et al., 2004). On the basis of pollen morphology, Harley, Paton, Harley, and Cade (1992) placed *O. gratissimum* and *O. tenuiflorum* in same group, while *O. basilicum*,* O. americanum*, and *O. kilimandscharicum* together in another group. On the other hand, *O. gratissimum* outgrouped in the *O. basilicum* cluster in a RAPD‐based analysis carried out by Shasany et al. (2007).

By contrast, in all the phylogenetic trees, grouping of *Ocimum* spp. based on ISSR and *psbA–trnH* sequence dataset differs largely. Neighbor‐joining analyses based on the ISSRs dataset placed “Gratissimum” group away from both “Sanctum” and “Basilicum” groups, while the distance‐ and character‐based analyses on the *psbA‐trnH* sequence dataset placed “Gratissimum” group with “Basilicum” group. The best explanation for this grouping is that the ISSRs (biparentally inherited) amplified from both nuclear and organellar regions, while *psbA‐trnH* region amplified from cpDNA region (maternally inherited). The size of the nucleome in comparison with the plastome and the chondriome is large. Hence, the origin of the ISSR bands is likely to be nuclear and can serve as control for the groups found with the plastid region. Due to haploid and uniparental inheritance, cpDNA reveals only half of the parentage of plants with hybridization/introgression or polyploidy and may incorrectly categorize plants into a clade of one of the two parents. Therefore, phylogenetic trees based on cpDNA may be discordant (Treutlein, Smith, van Wyk, & Wink, 2003). The present report indicated the high efficiency of ISSRs markers in resolving the taxonomy of *Ocimum*. Interestingly, the earlier study by Christina and Annamalai (2014) proved that *psbA–trnH* region was a more suitable barcode as compared to the other tested cpDNA regions (*matK*,* rbcL*) for *Ocimum* species identification, but not for differentiating their varieties. They also indicated that the study of deletions in the sequences could prove helpful in species identification. Moreover, study carried out by Bhamra, Heinrich, Howard, Johnson, and Slater (2015) in *Ocimum* illustrated *psbA‐trnH* region to be more efficient in species identification than nuclear ribosomal internal transcribed spacer (*ITS*).

ISSR technique is a PCR‐based method with advantages of high sensitivity to low levels of genetic variation, low‐cost, and high efficiency as compared with other DNA genotyping techniques, thus making it a very useful molecular tool for studying population genetics on a wide range of plant species, as well as for identifying species, or cultivars (Wang, Feng, Lu, Shi, & Liu, 2009). The present phylogeny study on *Ocimum* based on ISSR markers strongly supported genetic divergence and also correlated to some extent with the morphological level.

To date, various molecular marker techniques have been developed and are used in phylogenetic investigations. Although ISSR markers are highly efficient and reproducible, yet they have not been used extensively in phylogenetic studies. Many phylogeny studies carried out through ISSRs as in case of *Vigna* (Ajibade, Weeden, & Chite, 2000), Asian‐cultivated rice *Oryza sativa* (Joshi, Gupta, Aggarwal, Ranjekar, & Brar, 2000), wheat (Nagaoka & Ogihara, 1997), and finger millet (Salimath, Oliveira, Godwin, & Bennetzen, 1995) have been found to be very effective. This study provided valuable information for the potential application of ISSR markers in phylogenetic investigation, and there is boundless scope to use this powerful technique in resolving intra‐ or interspecific status in many genera or in deciding the uniqueness of different genera within a family. Along with the vast applications of ISSR markers, unique alleles produced in the present study for *Ocimum* can be converted in codominant sequence characterized amplified regions (SCARs) to develop species‐specific diagnostic markers for a fast and effective diagnosis of this important medicinal material.

## Conflict of Interest

The authors declare that they have no competing interests.
